# Breaking down barriers in medical education: addressing racism and discrimination

**DOI:** 10.1097/JS9.0000000000000312

**Published:** 2023-03-14

**Authors:** Olivier Uwishema

**Affiliations:** Oli Health Magazine Organization, Research and Education, Kigali, Rwanda

*Dear Editor,*

The issue of racism and discrimination in medical education and its impact on healthcare has been a long-standing and pressing concern. Unfortunately, these forms of prejudice and bias persist in our healthcare system and medical education, even in today’s society^1^.

Racism and discrimination in medical schools can take many forms, such as discriminatory admissions procedures, unequal treatment of students and faculty, and a lack of diversity in the curriculum. This can lead to a culture in which students of color and other marginalized groups feel unsupported and isolated. Furthermore, it can create an environment in which these students are less likely to succeed and more likely to experience negative mental health outcomes^1^,^2^.

The effects of racism and discrimination in medical education extend beyond the students themselves, and have serious consequences for patients and the healthcare system as a whole. Medical students who experience prejudice and bias are more likely to have negative attitudes towards marginalized communities and, as a result, may provide substandard care. This can have a significant impact on the health and well-being of patients and can contribute to health disparities and unequal outcomes^3^,^4^.

To address this problem, it is essential that medical schools take a proactive approach to promoting diversity, equity, and inclusion. This includes implementing policies and programs that support students of color and other marginalized groups, as well as incorporating diversity into the curriculum and promoting culturally competent care. Furthermore, medical schools must foster an environment in which students and faculty feel supported and valued and where discussions about racism and discrimination are encouraged and welcomed^5^.

Aside from these efforts, medical schools must address the underlying causes of racism and discrimination in the healthcare system, such as implicit bias and systemic oppression. This can be accomplished through education and training programs that teach students, faculty, and staff how to recognize and challenge their biases^5^.

In conclusion, it is time for medical schools to speak out against racism and discrimination and work to create a healthcare system that is inclusive, equitable, and culturally competent. By doing so, we can ensure that all patients, regardless of their background or identity, receive the highest quality of care.

## Ethics approval

Not applicable.

## Consent

Not applicable.

## Sources of funding

The author have not received any financial support for this manuscript.

## Author’s contribution

O.U.: conceptualization, project administration, writing – review and designing, final draft.

## Conflicts of interest disclosure

The author declared no conflicts of interest.

## Guarantor

Olivier Uwishema: principal investigator (PI).

## Data availability statement

Not applicable.
